# Calcium Signaling in the Thyroid: Friend and Foe

**DOI:** 10.3390/cancers13091994

**Published:** 2021-04-21

**Authors:** Muhammad Yasir Asghar, Taru Lassila, Kid Törnquist

**Affiliations:** 1 Minerva Foundation Institute for Medical Research, Biomedicum Helsinki 2U, Tukholmankatu 8, 00290 Helsinki, Finland; yasir.asghar@helsinki.fi (M.Y.A.); taru.lassila@abo.fi (T.L.); 2 Cell Biology, Faculty of Science and Engineering, Åbo Akademi University, Artillerigatan 6, 00250 Turku, Finland

**Keywords:** thyroid, cancer, channels, receptors, signaling, calcium

## Abstract

**Simple Summary:**

All cells in our body are activated by several different signals. The calcium ion is one of the most versatile signaling molecules, and regulates a multitude of different events in the cells. These range from activation of muscle contraction, to the regulation of cell movement, just to name a few. In normal thyroid cells, calcium signaling is of importance for the normal physiology of the cells. In thyroid pathologies, e.g., thyroid cancer, calcium is important for the regulation of proliferation and invasion, and may also activate gene transcription programs important for cancer cell survival. In this Commentary, we summarize what is known regarding calcium in the normal thyroid, and highlight the importance of calcium signaling in thyroid pathologies.

**Abstract:**

Calcium signaling participates in a vast number of cellular processes, ranging from the regulation of muscle contraction, cell proliferation, and mitochondrial function, to the regulation of the membrane potential in cells. The actions of calcium signaling are, thus, of great physiological significance for the normal functioning of our cells. However, many of the processes that are regulated by calcium, including cell movement and proliferation, are important in the progression of cancer. In the normal thyroid, calcium signaling plays an important role, and evidence is also being gathered showing that calcium signaling participates in the progression of thyroid cancer. This review will summarize what we know in regard to calcium signaling in the normal thyroid as, well as in thyroid cancer.

## 1. Introduction

Perhaps the most versatile cellular signaling molecule is the calcium ion. The mechanisms by which calcium signaling occurs in cells are very disperse. Several cellular signaling pathways evoke potent changes in intracellular calcium concentrations through different pathways. One extremely important pathway, both from a physiological and clinical point of view, is the activation of G protein coupled receptors. Phospholipase C can become activated both through the activation of Gi and Gq, resulting in the breakdown of phosphoinositol bisphoshate (PIP_2_) to inositol trisphosphate (IP_3_) and dicaylglycerol. IP_3_ diffuses through the cytosol and activates IP_3_ receptors on the endoplasmic reticulum (ER), resulting in a rapid and transient increase in cytosolic free calcium concentrations ([Ca^2+^]i). Depending on the intensity of stimulation, the IP_3_-evoked changes in [Ca^2+^]_i_ result in rapid oscillation and the vastly different frequencies of these oscillations result in a multitude of downstream signals. The produced DAG, in turn, can activate different types of calcium channels in the plasma membrane, depending on the cell type. Interestingly, experimental evidence has also shown that arachidonic acid-regulated calcium-selective (ARC) channels are of importance for oscillatory calcium entry when cells are stimulated with low agonist concentrations. This response is, thus, not linked to the release of ER calcium stores. The PLC-IP_3_ signaling pathway can also be activated by tyrosine kinase receptors. As a prolonged increase in cytosolic calcium is poisonous, the calcium entering the cytosol is pumped out of the cell by plasma membrane calcium ATPAses (PMCAs), and back to the ER by different types of sarcoplasmic-endoplasmic calcium ATPAses (SERCAs) in the ER membrane. In particular, in excitable cells, the sodium–calcium exchange through the Na^+^/Ca^+^ exchanger (NCX) also transports calcium out of the cells but may also participate in non-excitable cells. The calcium signal can result in the activation of gene transcription, energy production in the mitochondria, regulate membrane potential, or evoke proliferation and migration of the cell, just to name a few examples (for excellent reviews, see [1,2,3,4]).

**Figure 1:**
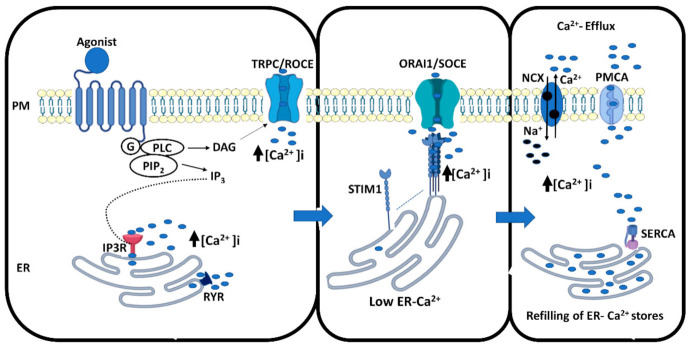
Mechanisms of cellular calcium signaling. A hormone or ligand binds to the G protein-coupled receptor and activates G-protein subunits Gi, Gq (G) or both, which activates phospholipase C (PLC), stimulating production of two secondary messengers; diacylglycerol (DAG) and inositol 1,4,5-trisphosphate (IP3). DAG potentially stimulates TRPC calcium channels in the plasma membrane and triggers calcium influx into the cells, referred to as receptor-operated calcium entry. IP3 binds to the IP_3_ receptors present on the endoplasmic reticulum (ER) membranes. This leads to the release of calcium from ER stores. These mechanisms rapidly increase cytosolic free calcium concentrations ([Ca^2+^]_i_). The depletion of the ER is sensed by STIM1 molecules on the ER membrane, which rapidly tetramerize and bind to ORAI1 calcium channels on the plasma membrane, resulting in the opening of these channels and a huge influx of calcium into the cells, referred to as store-operated calcium entry. To avoid an overload of ([Ca^2+^]_i_) in the cytoplasm, the pumps SERCA, NCX and PMCA, are activated and calcium is shuttled back into the ER to refill the stores, or exported out from the cells, respectively. The events described are shown in three different panels for clarity.

In addition to the above described receptor-evoked changes in [Ca^2+^]_i_, the emptying of the ER induces store-operated calcium entry (SOCE) [5]. This is the result of the tetramerization of the ER calcium sensor stromal interacting molecule 1 (STIM1), followed by diffusion along the ER membrane and coupling with the plasma membrane Orai calcium channels (Figure 1). This results in a rapid entry of extracellular calcium into the cytosol, and refilling of the ER calcium store through pumping by the sarcoplasmic-endoplasmic calcium ATPase (SERCA). It is also of importance to note that Orai isoforms, and plasma membrane-residing STIM1 molecules are needed for forming the ARC channels [4].

**Figure 2:**
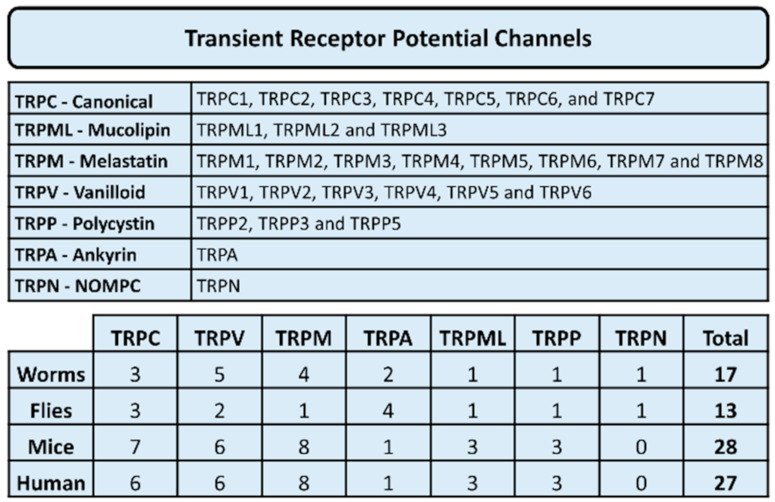
Classification of transient receptor potential ion channels.

In addition to coupling to the Orai proteins, STIM1 can also couple to members of the transient receptor potential (TRP) family of ion channels, in particular, to members of the TRPC subfamily [6]. Transient receptor potential (TRP) channels are a large family of cation channels (Figure 2). The expression of these calcium channels is diverse and has been identified in different animal species, including humans. These channels are classified into seven subgroups of TRP channels, i.e., TRPC (canonical), TRPM (melastatin), TRPV (vanilloid), TRPML (mucolipin), TRPA (ankyrin), TRPP (polycystin) and TRPN (no mechanoreceptor potential C). In humans, all other TRP channel subgroups have been detected except for TRPN. These calcium channels have been found to regulate several cellular processes including fluid secretion, pain, inflammation, heat sensation, odor and smell, cell adhesion, proliferation, migration and invasion and cell death in both healthy and diseased cells. For a thorough review on the importance and diversity of the TRP family of cation channels, see [7].

In addition to the TRP channels, cells can also express different types of voltage-dependent channels (VOCCs). In non-excitable cells, such as the thyroid cells, the VOCCs are of less importance (but might be expressed). Calcium can also be released from the ER and the sarcoplasmic reticulum through ryanodine receptors by calcium-induced calcium release. However, as for VOCCS, these channels are mostly expressed in excitable cells. In muscle and neuronal cells, VOCCs and ryanodine receptors are of crucial importance for their function [1].

The aim of this review is to provide an overview of how calcium ions participate in regulating the normal function of the thyroid follicular cells, and how disturbances in calcium signaling might be of importance in thyroid cancer.

## 2. Thyroid and Calcium Signaling

Traditionally, TSH-evoked activation of the cyclic AMP (cAMP) pathway has been considered to be the most important signaling pathway in the thyroid [8]. However, a vast number of investigations have clearly shown that calcium signaling in the thyroid has a surprisingly important role. Using rat thyroid FRTL-5 cells, several laboratories have made a multitude of investigations. These studies have shown that, e.g., both the proliferation and DNA synthesis is crucially dependent on calcium signaling [9,10,11,12]. We showed, that the TRPC2 ion channel, a member of the TRPC family, was needed for proliferation of FRTL-5 cells [12]. Although TRPC2 is a pseudogene in humans, our observation is the first to show that the TRPC family of calcium channels is of importance in the thyroid. The fact that only the TRPC2 ion channel of the TRPC family was expressed in the FRTL-5 cells made it easy to dissect the importance of the channel for different functions in the cells. TRPC2 seems to be of importance in regulating the general calcium homeostasis in these cells, as well as stromal interacting molecule 2 (STIM2) [13]. Furthermore, several agonists, including ATP, UTP, sphingosine 1-phosphate (S1P), TSH, carbachol, and noradrenalin, evoke potent calcium signals in these cells [11,14,15,16,17,18,19]. Additionally, in human and dog thyroid cells, several agonists evoke calcium signals [20,21]. These agonist-evoked calcium signals are mediated by the PLC-IP_3_ pathway, but the FRTL-5 cells (at least) also show strong activation of the SOCE pathway [22]. Some results also suggest that, the FRTL-5 cells (at least), might express ARC channels, but the molecular mechanisms have not been determined in detail [23]. Taken together, agonist-evoked release of stored calcium, and SOCE participate in the regulation of [Ca^2+^]_i_ in thyroid cells. Furthermore, members of the TRPC family seem to participate in the regulation of calcium in thyroid cells.

As mentioned above, TSH can, in addition to activating the cAMP/PKA pathway, also evoke calcium signals, albeit at rather high concentrations. An amount of 0.3 mU TSH/mL results in strong activation of the cAMP/PKA pathway, whereas at least 10 mU TSH/mL, or even up to 100 mU/mL, depending on species, is needed to evoke a calcium signal [24]. Of note, cAMP, in turn, can attenuate agonist-evoked calcium signals, at least in the FRTL-5 cells [25,26]. It is still unclear how this effect is mediated, but the calcium-binding protein S100A4 seems to be involved [26]. cAMP also attenuated calcium signals in other cell types [27]. This is interesting, as cAMP has been shown to potently enhance agonist-evoked calcium signals through PKA-mediated phosphorylation of the IP_3_ receptors [28].

It is also interesting to note that the regulation of TSH receptor expression is dependent on calcium signaling [29]. In the FRTL-5 cells, we showed that the TRPC2 channel seems to have an important role in this regulation [30]. Furthermore, both the uptake and extrusion of iodide is regulated by calcium, as shown in both FRTL-5 cells, and dog and human thyroid slices [24,31,32,33,34,35]. The importance of the transport protein pendrin has been shown in this process ([36], but see [37]), but no information is presently available in regard to calcium and the function of pendrin. However, the anion channel anoctamin-1/TMEM16A has been shown to effectively enhance iodide efflux in a calcium-dependent manner [38,39,40]. In FRTL-5 cells, the TRPC2 channel is of importance in regulating anoctamin-1 function [38]. An interesting observation is that 3-iodothyronamine decreases the expression of genes involved in iodide metabolism and inhibits iodide uptake in PCCL3 thyroid cells [41]. It is possible that the TRPM8 ion channel and calcium signaling might be involved in this process, as 3-iodothyronamine appears to be an endogenous modulator of TRPM8 [42]. It has also been shown that the expression and dimerization of thyroglobulin is dependent on calcium [43], and that the DREAM (downstream regulatory element antagonist modulator) protein participates in this process [44]. Furthermore, the thyroid hormone synthesis, mediated by the H_2_O_2_ producing oxidase Duox, is dependent on calcium signals [45]. It is also worth mentioning that the TSH-evoked activation of the cAMP pathway can be modulated by calcium signaling, as the adenylyl cyclase isoforms V and Vl are negatively regulated by calcium, and these isoforms are found in both human, dog, and rat FRTL5 thyroid cells [30,46].

Both human thyroid cells, rat FRTL-5 cells and several human thyroid cancer cells express several calcium binding proteins belonging to the S100 class of calcium binding proteins. Interestingly, in both human thyroid cells in culture, and in FRTL-5 cells, TSH participates in the regulation of these proteins, i.e., S100A4 and S100A6 [26,47]. Furthermore, TSH-prestimulation of the cells attenuated the ATP-evoked calcium signaling, presumably through enhanced expression of, e.g., S100A4. These results were confirmed by either knock-down or overexpression of 100A4, and stimulation with ATP.

For a long time, the nature of calcium channels in thyroid cells was not well known. We previously showed that at least FRTL-5 cells expressed several isoforms of the P2X family of channels [11]. Some evidence also exists that these cells should express L-type voltage operated calcium channels [9,48]. We have not been able to repeat these studies. As the authors used, e.g., verapamil to block calcium fluxes in the FRTL-5 cells [9,48], and as verapamil also is a blocker of channels belonging to the TRP superfamily, the authors were probably the first to show that the cells express channels belonging to the TRP superfamily. When these reports were published, the TRPC channels had not yet been cloned. However, although VOCCs are mostly of importance in excitable cells and not in non-excitable cells [49], we cannot totally exclude the possibility that, e.g., human thyroid cells may express L- or T-type VOCCs, and that these might participate in the regulation of calcium homeostasis. Recent studies have clearly shown that thyroid cells express several members of the TRP superfamily. We have shown that the FRTL-5 cells express TRPC2 [13], while we and others have also shown that human thyroid cells express TRPC1, and TRPC3-6 [50,51]. Furthermore, the channels TRPV1 [52] and TRPV6 [53] are also expressed.

In addition to these channels participating in calcium entry in thyroid cells, release of stored calcium from the ER results in store-operated calcium entry (SOCE). In FRTL-5 cells, we showed that emptying the ER with thapsigargin resulted in a robust influx of extracellular calcium [22]. As mentioned above, the FRTL-5 cells express both STIM1 and STIM2 (the calcium sensing proteins in the ER), and at least the Orai1 protein (which functions as calcium channels in the plasma membrane) [13], important proteins in the regulation of SOCE. Preliminary results from our laboratory show that, not unexpectedly, these proteins are also expressed human thyroid cells (Asghar et al., submitted).

## 3. Calcium Signaling in Thyroid Pathologies

The importance of calcium signaling in thyroid pathologies has not been extensively investigated. Mutations (I846F, 486M, I568T) causing constitutive activity of the TSH receptor activated the IP_3_-diacylglycerol pathway and resulted in hyperfunctioning thyroid adenomas [54]. Contrary to this, an L653V mutation strongly attenuated the TSH-evoked IP_3_-Ca^2+^ signaling pathway in the human thyroid [55]. Homozygous individuals with this mutation had euthyroid hyperthyrotropinemia. Furthermore, the DREAM protein has been associated with thyroid enlargement and thyroid nodule formation, but coupling to calcium signaling was not determined [56].

Calcium binding proteins of the S100A family have been involved in several forms of cancer, including thyroid cancer [57]. Of these proteins, especially S100A13 [58] and S100A4 [59,60], seem to be important for the proliferation, invasion and metastasis of thyroid cancer cells. Furthermore, knock-out of S100A4 has been shown to sensitize anaplastic thyroid carcinoma cells to vemurafenib, especially cells with the BRafV600E mutation [61]. An interesting observation is that anoctamin5, a member of the anoctamin/TMEM16 family of calcium-regulated chloride channels, seems to be important for thyroid cancer cell migration and invasion. Anoctamin 5 was downregulated in thyroid cancer specimens, and in vitro experiments showed that overexpression in cancer cells attenuated migration and invasion [62]. Furthermore, although the importance and possible expression of VOCCs in thyroid cells is still unclear, some evidence has been obtained for the expression of T-type voltage operated calcium channels in a medullary thyroid cancer cell line [63]. Thus, the possibility of VOCCs in thyroid cancer cells cannot be excluded [64].

One important regulator of cancer progression in many cell types, is calcium and different calcium channels [65,66]. Calcium signaling in thyroid cancer has, however, not been well studied. We have shown that the TRPC1 ion channel is of importance in regulating follicular thyroid cancer ML-1 cell proliferation, migration and invasion [50]. Our investigation showed that knocking down TRPC1 attenuated the expression of migratory VEGFR2 and S1P receptors, as well as attenuating the expression and secretion of MMP2 and -9. We have shown that both STIM1 and Orai1 are upregulated in thyroid cancer cells, as compared with normal thyroid cells, and are important regulators of thyroid cancer cell proliferation and migration (Asghar et al., submitted). Other investigations have indicated that the TRPV1 and TRPV6 ion channels are of importance in thyroid cancer cells. TRPV1 was shown to be upregulated in thyroid cancer [53]. In a recent report, Xu et al. showed that, in human papillary thyroid carcinoma BCPAP cells, activation of TRPV1 with capsaicin, attenuated both the migration and invasion of these cells [67]. In addition, activation of TRPV1 significantly attenuated the expression of MMP2 and 9. In a follow up study, the same Authors showed that capsaicin-evoked activation of TRPV1 induced calcium overload in the mitochondria, resulting in the apoptosis of anaplastic thyroid carcinoma cells [52]. Our unpublished work also suggests that, the transcription factors TRβ1 and RUNX2 may be regulated by calcium signaling (Lassila and Törnquist, manuscript in preparation).

Recently, a transient receptor potential channel 4-associated protein (TRPC4AP, also known as TNFα-receptor ubiquitous signaling and scaffolding protein, TRUSS) was suggested to be an important gene causing congenital primary hypothyroidism [51]. Knock-down of this gene in *Xenpus laevis* tadpoles resulted in a decrease in the thyroid anlage, and the authors suggested that TRPC4AP could be of importance in the regulation of thyroid cell proliferation and the expression of thyroid-specific proteins. The link to the regulation of calcium signaling seems strong, as previous experiments have shown that TRPC4AP interacts with members of the TRPC ion channel family and participates in the regulation of ER calcium stores [68].

## 4. Conclusions

It can be clearly stated that calcium signaling certainly has an important role in the normal physiology of the thyroid. However, the importance of different calcium channels and transporters in thyroid pathologies, including cancer, is still not well known. The expression of, e.g., many ion channels belonging to the TRP family of ion channels in the thyroid, as well as both STIM- and Orai proteins, strongly indicates that these may have an important role on the etiology of thyroid cancer, in a manner similar to cancers in other tissues (please see, e.g., [64]). The problem in regard to calcium channels, similarly to members of the TRPC family or either the STIM or Orai proteins, as targets for drug development, is their ubiquitous expression in many different tissues. A possibility could be the use of functionalized, cancer cell targeted nanoparticles, carrying suitable inhibitors. Such an approach has proven to be effective in mice with breast cancer [69], and we have shown that thyroid cancer cells effectively take up nanoparticles carrying metothrexate in vitro, inducing cell death of the cancer cells [57,70]. Furthermore, siRNA loaded nanoparticles for knock-down of channel proteins are also an attractive option [71]. We have shown that these can effectively be used for thyroid cancer cells, at least in vitro (Asghar et al., submitted). However, it is clear that for using these approaches, more research is needed to optimize an effective treatment strategy for clinical use.
